# A global daily evapotranspiration deficit index dataset for quantifying drought severity from 1979 to 2022

**DOI:** 10.1038/s41597-023-02756-1

**Published:** 2023-11-24

**Authors:** Xia Zhang, Jianping Duan, Francesco Cherubini, Zhuguo Ma

**Affiliations:** 1 Industrial Ecology Programme, Department of Energy and Process Engineering, Norwegian University of Science and Technology (NTNU), 7491 Trondheim, Norway; 2 Key Laboratory of Regional Climate-Environment Research for Temperate East Asia, Institute of Atmospheric Physics, Chinese Academy of Sciences, 100029 Beijing, China; 3 State Key Laboratory of Earth Surface and Ecological Resources, Faculty of Geographical Science, Beijing Normal University, 100875 Beijing, China; 4 University of Chinese Academy of Sciences, 101408 Beijing, China; 5 Xiongan Institute of Innovation, Chinese Academy of Sciences, 071899 Xiongan New Area, China

**Keywords:** Natural hazards, Environmental health

## Abstract

Droughts cause multiple ecological and social damages. Drought indices are key tools to quantify drought severity, but they are mainly limited to timescales of monthly or longer. However, shorter-timescale (e.g., daily) drought indices enable more accurate identification of drought characteristics (e.g., onset and cessation time) and help timely potential mitigation of adverse effects. Here, we propose a dataset of a daily drought index named daily evapotranspiration deficit index (DEDI), which is produced for global land areas from 1979 to 2022 using actual and potential evapotranspiration data. Validation efforts show that the DEDI dataset can well identify dry and wet variations in terms of spatial patterns and temporal evolutions when compared with other available drought indices on a daily scale. The dataset also has the capability to capture recent drying trends and to detect ecology- or agriculture-related droughts. Overall, the DEDI dataset is a step forward in facilitating drought monitoring and early warning at higher temporal resolution than other compared existing products.

## Background & Summary

Drought occurs in the presence of water scarcity, and it is characterized by a lack of precipitation that induces an imbalance between moisture supply and demand^1–3^. Drought has caused enormous ecological damages and socioeconomic losses around the globe, which is under increasing public concern^2,4–6^. The major drought events in Africa in the mid-1980s led to a widespread famine with nearly one million deaths^7^. In India, a serious drought affected 300 million people in 2002 with water shortages and food scarcity^8^, and in 2006 a drought caused an estimated $3.5 billion of economic losses in Australia^9^. In China, the direct economic losses resulted from drought events exceeded an average of ¥44 billion per year^10^. This evidence emphasizes the critical importance and need of drought monitoring and early warning. However, drought is a complicated phenomenon^7,11,12^ and has a multi-timescale nature^13–15^. Most of drought studies focus on monthly or seasonal scales^11,16,17^, and the lack of daily-scale drought metrics poses great challenges to accurately identify the onset and cessation time, intensity evolution, and spatial extent^18^.

Within this context, drought indices have been developed as a powerful tool by synthesizing the key factors, and they have been used to quantify the impacts of drought on ecological and socioeconomic sectors^1,19–21^. Despite the numerous existing drought indices, these indicators present different regional applicability when used in various scenarios (e.g., meteorological, hydrological, agricultural or ecological, and socioeconomic droughts)^16,22,23^. Widely used indices such as the standardized precipitation index^13^ or the standardized precipitation evapotranspiration index (SPEI)^20^ are generally based on a monthly timescale and cannot identify the onset and termination days of droughts. By contrast, drought indices with shorter timescales (e.g., daily) are scarcer. This is a main drawback as there are risks that droughts with short duration (e.g., several days), such as flash drought^24^, remain undetected. Flash drought has increasingly occurred under ongoing global warming and caused substantial crop yield reductions by impacting critical plant growth stages including germination, pollination, and grain filling^24–26^.

**Fig. 1:**
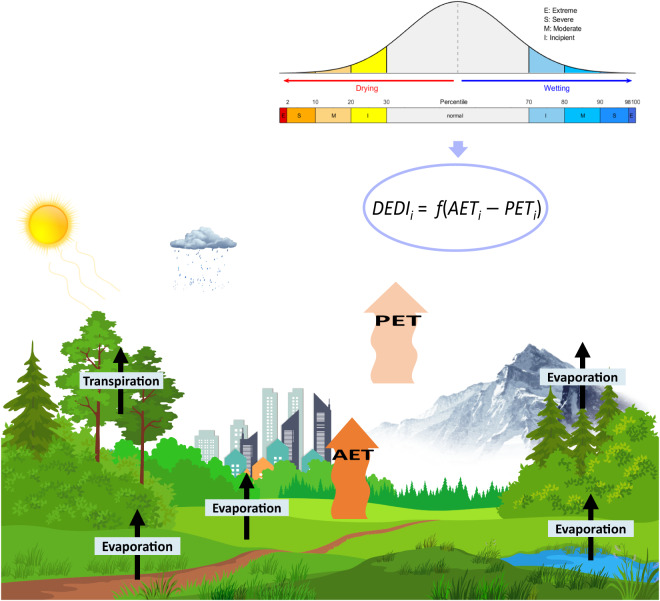
Schematic diagram of the physical processes involved in calculation of the Daily Evapotranspiration Deficit Index (DEDI). The upper right panel shows the DEDI formula calculated by actual evapotranspiration (AET) and potential evapotranspiration (PET) and the corresponding thresholds in different dry and wet intensities. AET involves surface (e.g., vegetation, ground, and water) evaporation and vegetation transpiration, and PET indicates the evaporative demand of the atmosphere. Extreme drought, severe drought, moderate drought, and incipient drought have a 2% or less, 2–10%, 10–20%, and 20–30% chance of occurring at any given location in any given time, respectively. The thresholds for humid categories are greater than 70% and the interval between the intensities is similar as the dry categories.

To cover this knowledge gap, a daily drought index named daily evapotranspiration deficit index (DEDI)^27^ has been recently developed to monitor short-duration droughts. DEDI has been shown to reasonably identify onset and cessation time of serious drought events^27^. However, existing work is confined to verify the performance of DEDI in detecting and forecasting short-duration regional drought events over China^27,28^, and the potential of DEDI for drought detection at a global level and under a long-term climatology has not been explored so far. Furthermore, DEDI considers actual evapotranspiration (AET) and potential evapotranspiration (PET), which combines the actual water supply from land surface and the maximum possible evaporation of the atmosphere^1,27,29,30^. As shown by the schematic diagram in Fig. 1, DEDI is based on the balance between the atmospheric evaporation demand and the actual land water evaporated from vegetation, soil, and water surfaces and transpired from vegetation into the atmosphere, driven by solar radiation energy. As such, DEDI involves water and energy cycle processes and connects the climate system with terrestrial ecosystems. Thus, the availability of a DEDI dataset with a global coverage is a novel contribution to better quantify different degrees of drought severity, as DEDI can provide relatively accurate identification of the onset and cessation time of drought events (so helping the deployment of possible mitigation measures) and has the potential to be a useful indicator to detect ecology- or agriculture-related droughts.

The primary aim of this study is to provide a robust daily drought index (i.e., DEDI) dataset with a long time series (1979–2022) covering global land areas. To provide a comprehensive assessment of the proposed DEDI dataset, we first verify its reliability against current widely accepted drought indices reprojected on a daily timescale, then characterize its performance in capturing drying trends reflected by daily drought signals, and finally identify its capability of detecting ecology- or agriculture-related droughts. The results are expected to confirm the robustness of the DEDI dataset and promote its use by global users interested in drought dynamics, impacts, and mitigation.

## Methods

### Data sources

We produced the DEDI dataset over global land areas using the daily AET and PET data during the period 1979–2022 provided by the European Centre for Medium-Range Weather Forecasts (ECMWF) Reanalysis v5 (ERA5)^31^. ERA5 products^32^ are the state-of-the-art reanalysis developed by ECMWF and are updated daily with a latency of 5 days. ERA5 data can well depict energy and water cycles because of the incorporation of high-quality meteorological observations and the good representation of land surface processes^33–35^. ERA5 uses hydrology tiled ECMWF scheme for surface exchanges over land (HTESSEL) land surface scheme to calculate land components^32^. The AET of ERA5 is treated as the accumulated water amount over a particular time period, including bare ground, vegetated surface, and water evaporation as well as vegetation transpiration. The PET is calculated as the maximum possible evaporation based on surface energy balance assuming a vegetation type of crops and no soil moisture stress. The ECMWF Integrated Forecasting System convention specifies that downward fluxes are positive, i.e., negative values indicate evaporation and positive values indicate condensation. More details can be found from the online ERA5 documentation (https://confluence.ecmwf.int/display/CKB/ERA5%3A+data+documentation). In addition to AET and PET, precipitation, 2 m temperature, 2 m dewpoint temperature, and soil moisture at a daily interval provided by ECMWF were employed to derive or directly serve as other reference indicators. We selected surface soil moisture in the top layer (0–7 cm), because surface soil moisture anomalies can propagate and influence dynamics throughout the soil profile and thus signal the change in drought conditions and affect ecosystem dynamics^36,37^. The dataset used for surface soil moisture is from ERA5-Land reanalysis^38^, which has been demonstrated to have added value from the enhanced hydrological cycle^39^. As all variables from ERA5 have a spatial resolution of 0.25°, the soil moisture data from ERA5-Land at a 0.1° resolution were aggregated at a 0.25° spatial resolution to match the grid size of the drought indices.

The Global Land Evaporation Amsterdam Model version 3.8a (GLEAM)^40^ datasets on a 0.25° latitude-longitude regular grid and at a daily temporal resolution provides PET and AET data from 1980 to 2022. GLEAM datasets are regularly updated and extended once a year. Previous studies have intensively proved the relatively high quality of the GLEAM data in hydrometeorology fields^29,41,42^. Hence, the GLEAM datasets were selected to help assess the influences of using different data sources on the DEDI calculations. GLEAM estimates PET according to observed surface net radiation and air temperature using the Priestley and Taylor equation. The AET is converted from the PET using a multiplicative evaporative stress factor based on microwave vegetation optical depth observations and root-zone soil moisture estimates. Thus, the AET and PET values of GLEAM are quite similar at high latitudes where they are primarily covered with snow, which consequently induces several missing values in the DEDI calculations. Furthermore, the AET of GLEAM^40^ is regarded as the combination of the different components, i.e., bare-soil evaporation, open-water evaporation, transpiration, interception loss, and sublimation. The interception loss which is independent from soil water content should be carefully considered when comparing GLEAM AET with eddy-covariance measurements due to the discrepancies about the sources of energy driving this flux in nature^43,44^. This implies potentially poor reliability of the calculated DEDI values during rain events.

To show the performance of the DEDI in different climate zones, an analysis of DEDI based on the Köppen-Geiger climate classification was performed. The Köppen-Geiger climate classification dataset^45^ with a spatial resolution of 0.5° × 0.5° was developed using the long-term average climate (1901–2010) of global temperature and precipitation observations. To enable its consistency with the spatial resolution of the drought indices, we interpolated the Köppen-Geiger climate classification dataset into a 0.25° × 0.25° resolution using the nearest neighbor interpolation method.

### Daily DEDI calculations

A global high-resolution daily drought index (DEDI) dataset was prepared following previous work that was confined to characterize regional drought events over China^27,28^.

DEDI is computed under the consideration of water deficit by integrating actual water supply and atmospheric evaporation demand. The DEDI value for the day *i* is obtained by normalizing the water deficit value as:

1 $${D}_{i}=AE{T}_{i}-PE{T}_{i},$$

2 $$DED{I}_{i}=\frac{{D}_{i}-{D}_{i,ave}}{{D}_{i,std}}$$

where *D*_*i*_ denotes the evapotranspiration deficit between AET and PET on day *i*, and *D*_*i,ave*_ and *D*_*i,std*_ are the multi-year climatic mean and standard deviation, respectively. *DEDI*_*i*_ refers to the deviation of the current day evapotranspiration deficit from a long-term climatology (at least 30 years) for the same day of the years. In this study, the climatological normal period of 1981–2010 was used as a baseline to standardize the evapotranspiration deficit at each grid cell to generate the DEDI. Previous work^27^ has demonstrated that the choice of the time period (e.g., the full period and the 30-year subperiods) to calculate the climatology of evapotranspiration deficit has little effect on DEDI values. This 30-year-climatology-based approach facilitates instant calculation and provides near real-time drought information. However, climatological standard normals^46^ are identified in a changing climate and had better to be updated as time proceeds (usually on a decade level).

The classification categories of DEDI to depict dry and wet conditions (right top corner in Fig. 1) are determined by referring to a percentile approach^47^. DEDI values varying from negative to positive indicate dry to wet evolution, and larger absolute values of DEDI represent stronger dry or wet intensities. Through the percentile category approach, slightly dry conditions have a 21–30% chance of occurring at a given location in any given time, while moderate, severe, and extreme droughts occur 11–20%, 3–10%, and 2% or less, respectively. Based on the DEDI series during the climatology period of 1981–2010, the dry and wet categories of the DEDI at each grid cell were determined separately due to the heterogeneity of topography and climate change across grids, with a general value range of ±2.

For the calculation of DEDI, unrealistic ERA5 PET values can potentially occur in arid conditions due to excessive evaporation forced by dry air, according to the caveat of the official data provider. The unrealistic ERA5 PET values can propagate into DEDI and may lead to stronger drought magnitudes identified in arid conditions. Additionally, the PET computation over forests and deserts probably has some limitations due to the influence of the model structure^39^. This means that the interpretation of the DEDI should be cautious in certain contexts^32,39,40,48^, such as desert areas (e.g., the Sahara Desert) due to a lack of observations, forested regions (e.g., the Congo and Amazon basins) due to the ignorance of the signal under vegetation canopy, or frozen conditions (especially at high latitudes) due to the underrepresentation of freeze-thaw parameterization. These issues are not negligible in most of existing models including the models used in producing ERA5 and GLEAM data.

### Evaluation criteria

The reliability of the DEDI dataset was tested through comparisons with other five indicators, including SPEI, evaporative demand drought index (EDDI), the GLEAM-based DEDI (abbreviated as DEDI_GLEAM), soil moisture, and vapor pressure deficit. SPEI and EDDI are among the two most widely accepted drought indices and were chosen to verify how the DEDI dataset captures dry and wet variations. DEDI_GLEAM was used to measure the effects of different data sources on the DEDI calculations. Soil moisture and vapor pressure deficit were employed to investigate the performance of the DEDI dataset in identifying ecology- or agriculture-related droughts. Vapor pressure deficit was calculated as the difference between actual water vapor pressure and saturation water vapor pressure using ERA5 datasets of 2 m temperature and 2 m dewpoint temperature, with drier air as the value decreases.

SPEI^20^, which incorporates precipitation and PET to represent the climatic water balance, has been commonly used in drought detection^17,49–51^. SPEI value is calculated based on the deficit between precipitation and PET and then is normalized after fitting the cumulative sequences to the log-logistic probability distribution. SPEI value usually varies from −2 to 2, with lower-than-zero denoting dry conditions and greater-than-zero denoting wet conditions.

EDDI^1^, solely based on PET, incorporates anomalous atmospheric evaporative demand to offer early warning of agricultural drought, hydrologic drought, and fire-weather risk. EDDI is currently used as a drought monitoring and early warning guidance tool and provides near-real-time drought information (https://psl.noaa.gov/EDDI/). EDDI datasets are typically generated at 1-week through 12-month timescales. EDDI value is calculated from a nonparametric approach and adopts an inverse normal approximation to derive empirical probabilities. Positive EDDI values indicate wet anomalies, and negative values indicate drier-than-normal conditions, with enhanced drought intensities as value decreases. EDDI values have a range of around ±2.09.

Many drought indices^1,20^, including SPEI and EDDI, were built from the perspective of cumulative effects of long-term water scarcity considering drought as a slow-evolving hazardous event^3,19^. This is unfavorable for the analysis of the onset and cessation time of drought as well as flash drought events because, for instance, the available datasets of SPEI and EDDI are at weekly to monthly timescales. To enable the comparisons between the DEDI and the reference drought indices, we used ERA5 datasets to calculate the daily values of SPEI and EDDI as their original datasets are not available on a daily timescale. The detailed calculations refer to the original literature of SPEI^20^ and EDDI^1^.

### Statistical methods

To conduct the validation of the DEDI dataset, the commonly used statistics including Pearson’s correlation coefficient, root mean square error, and ordinary least squares linear trend were adopted. The correlation coefficient was calculated using the data series with the subtracted climatological averages, which could offer more rigorous evaluations regarding the relationship between the drought variations captured by drought indices^52^. Ordinary least squares linear trend was used to present the capability of the DEDI dataset to capture drying trends by detecting daily drought signals. The statistical significance was tested using a Student’s *t*-test.

In addition, we performed the spatial analysis including grid cells and regional scales as well as the temporal analysis by distinguishing the behaviours of the DEDI in different seasons. Regional analysis was conducted in terms of continental scales and climate zones, which could provide the information of the DEDI performance to users with different needs of targeted areas under diverse geographic and climatic conditions. Area ranges of the global continental analysis include global land (60 °S–90 °N), Asia (10 °N–80 °N, 25 °E–140 °E), Europe (30 °N–80 °N, 15 °W–70 °E), Africa (40 °S–40 °N, 20 °W–60 °E), Oceania (50 °S–0, 110 °E–160 °E), North America (10 °N–80 °N, 170 °W–50 °W), and South America (60 °S–20 °N, 100 °W–30 °W). Five major Köppen-Geiger climate classification types^45^ were used to determine global climate zones in this study, i.e., tropical climates, dry climates, mild temperate, snow, and polar. For the seasonal variations, all regions involved in this study were processed with their local time and then statistical analyses were performed, considering that the southern hemisphere has opposite seasons to the northern hemisphere. The seasonal analysis is from 1979 to 2021 except for the analysis related to the DEDI_GLEAM due to the availability of the dataset only since 1980.

## Data Records

The daily drought index DEDI dataset has been archived in the online open-access repository Zenodo^53^, available at 10.5281/zenodo.7768534. The DEDI dataset is derived from the AET and PET of ERA5 reanalysis data provided by ECMWF. The high-resolution dataset covers global land and has a spatial resolution of 0.25° latitude by 0.25° longitude for the period 1979–2022.

Given the large size of the high-resolution DEDI dataset, we have stored the index data year by year. Ultimately, the dataset contains 44 files, and each file is in a 3-D netCDF4 format (https://docs.unidata.ucar.edu/netcdf-c/current/index.html). Each file is labelled as “ERA5_DEDI_global_land_year_daily.nc” and has an array of 365 (time in daily, and 366 for leap years) × 721 (latitude) × 1440 (longitude). NetCDF4 data can be manipulated or visualized with open source software including Python, Climate Data Operators, Panoply, and Ncview (https://www.unidata.ucar.edu/software/netcdf/software.html). In addition, to reduce the pressure on users to download the dataset, we have compressed the data into a half of the original size using netCDF operators (NCO) version 4.9.5 (http://nco.sourceforge.net/). Consequently, the size of each netCDF4 file is around 520 MB, and the total size for the period 1979–2022 occupies 23 GB of space.

## Technical Validation

Here, we mainly present the technical validation of the DEDI dataset from the following three aspects: the reliability in identifying dry and wet variations, the skill in capturing recent drying trends, and the performance in characterizing ecology- or agriculture-related droughts.

### Relative evaluation of DEDI in identifying dry and wet variations

**Fig. 2:**
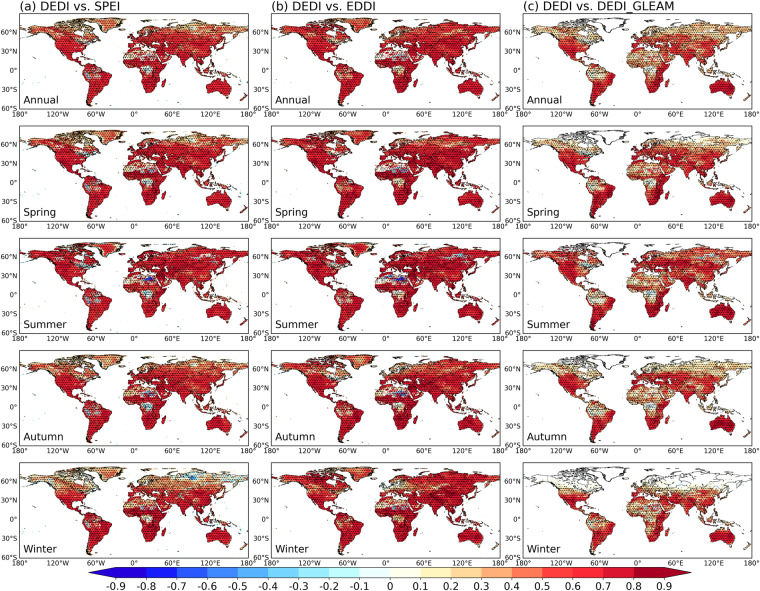
Spatial patterns of the correlation coefficients between the DEDI dataset and other reference daily drought indices (i.e., SPEI in column (**a**), EDDI in column (**b**), and DEDI_GLEAM in column (**c**)) in different seasons during the period 1979–2021. DEDI, SPEI, and EDDI denote daily evapotranspiration deficit index, standardized precipitation evapotranspiration index, and evaporative demand drought index, respectively, and DEDI_GLEAM denotes that the DEDI is calculated using GLEAMv3.8a data. The stippled areas indicate the correlation being statistically significant at a 5% level. GLEAMv3.8a datasets are only available since 1980, and thus the correlation coefficients between DEDI and DEDI_GLEAM refer to the time period 1980–2021 only.

A direct comparison between the DEDI dataset and the widely used drought indices over global land in different seasons is shown in Fig. 2, where the daily series of each pixel from different indices are correlated. The DEDI series generally have significantly high correlation coefficients (*p < *0.05) with the SPEI and the EDDI series in most areas of global land in all seasons, suggesting that DEDI has a similar ability to other reference indices in characterizing dry and wet variations. In winter, the lower consistency between the drought indices is probably due to freeze-thaw processes^54,55^.

The comparison between the DEDI dataset and the DEDI_GLEAM dataset provides information of how different data sources can influence DEDI calculations. The treatment of different models and assimilations employed in ERA5^32^ and GLEAM^40^ data makes distinct evapotranspiration values, which subsequently affects the magnitude of DEDI values. However, in general, the DEDI dataset can be highly correlated with the DEDI_GLEAM, with most grids being statistically significant at a 5% level. GLEAM produces approximately identical values between AET and PET at high latitudes, which makes some missing values in the DEDI_GLEAM, especially in winter. This occurs because AET in GLEAM is derived from PET and vegetation stress factor, and AET is equal to PET primarily in snow covered areas where vegetation stress factor is one^40^.

**Fig. 3:**
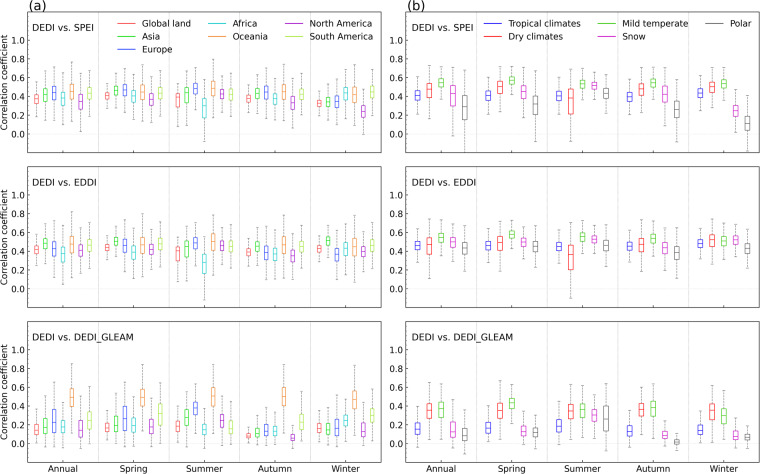
Comparisons of the correlation coefficients between the daily DEDI series and the reference daily drought indices in each grid of the column (**a**) global continental land areas, and the column (**b**) the Köppen-Geiger climate zones in different seasons during the period 1979–2021. The reference drought indices include SPEI, EDDI, and the DEDI_GLEAM. The box contains the values from the 25^th^ (bottom) to the 75^th^ (top) percentile, a horizontal line inside the box denotes the median, and the vertical lines denoting whiskers represent the extreme values. The correlation coefficients between DEDI and DEDI_GLEAM only refer to their common time period 1980–2021.

Figure 3 further shows the daily correlation series of the spatial patterns between the DEDI dataset and the reference drought indices over global continents and climate zones in different seasons. The DEDI shows positive correspondence with the reference drought indices, with statistically significant correlation coefficients passing a 5% level. The DEDI and the SPEI are correlated with median correlation coefficients greater than 0.4 for all seasons except winter when there is a slightly lower correlation. The DEDI over Asia, Europe, Oceania, and South America has consistently good agreement with the SPEI, but less agreement over Africa and North America. The DEDI achieves a higher consistency with the EDDI, with median correlation coefficients greater than 0.45. This occurs primarily because the two indices are both dominated by evapotranspiration. The DEDI dataset agrees well with the DEDI_GLEAM and attains the highest median correlations (greater than 0.5) over Oceania. From the perspective of climate zones, DEDI has the consistently best agreement with reference drought indices over mild temperate zones, followed by the zones with dry climates and tropical climates. Snow and polar zones present poor consistency between drought indices. This occurs largely because the limited data accuracy is resulted from the lack of observations and the underrepresentation of the freeze-thaw parameterization^32,39,40^.

**Fig. 4:**
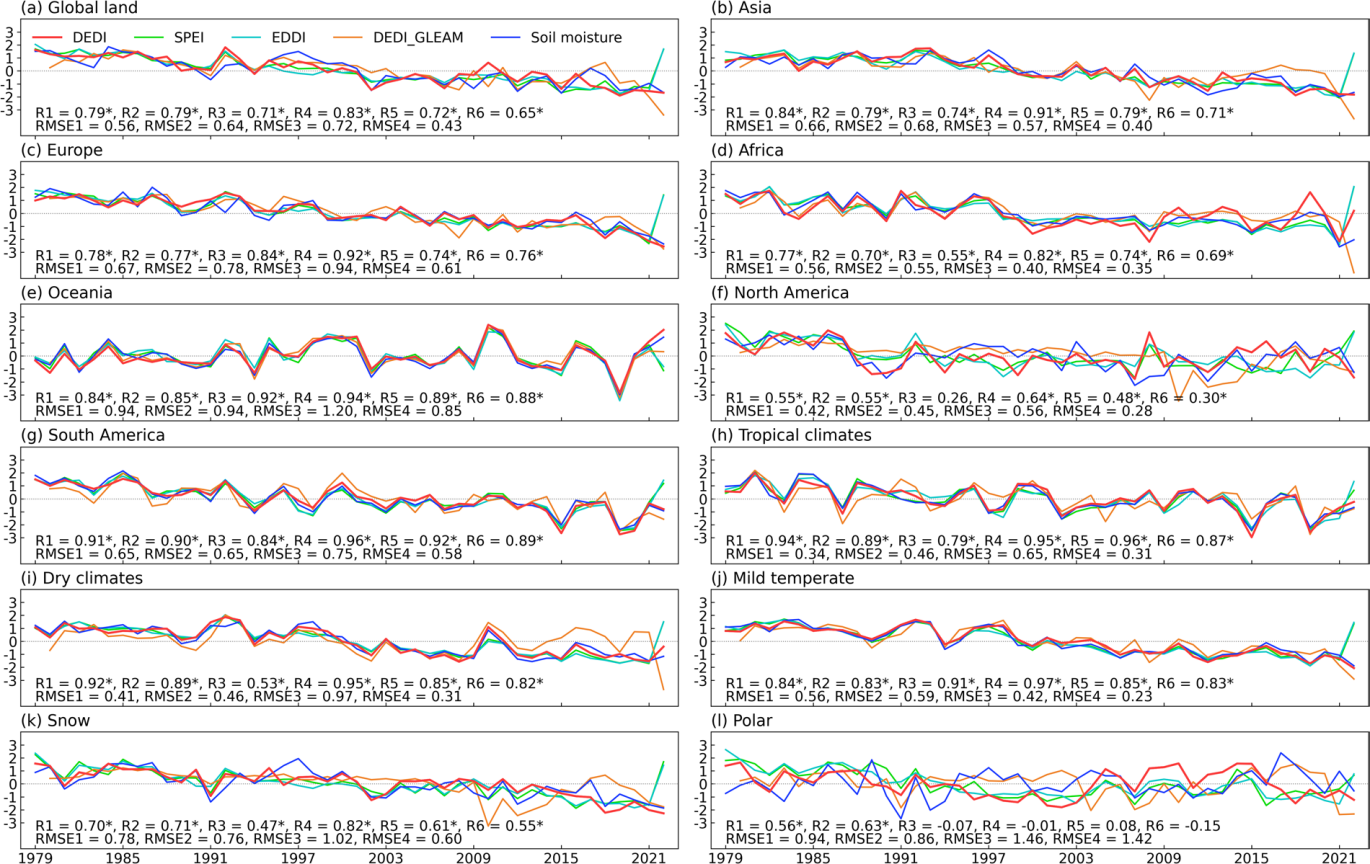
Annual variations of the DEDI with the reference drought indices and soil moisture averaged over (**a**) the global land, (**b**–**g**) six continents, and (**h**–**l**) five major Köppen-Geiger climate zones during 1979–2022. Annual value was averaged from the daily values of the growing season (i.e., March to August). The correlation coefficients with asterisks listed at the bottom of each panel indicate significance at a 5% level. R1, R2, and R3 denote the correlation coefficients between DEDI and SPEI, EDDI, and the DEDI_GLEAM, respectively. R4, R5, and R6 denote the correlation coefficients between soil moisture and DEDI, SPEI, and EDDI, respectively. RMSE1, RMSE2, RMSE3, and RMSE4 denote the root mean square errors between DEDI and SPEI, EDDI, the DEDI_GLEAM, and soil moisture, respectively. The correlation coefficients and RMSEs between DEDI and DEDI_GLEAM only refer to their common time period 1980–2022. All series are normalized for visual clarity.

**Fig. 5:**
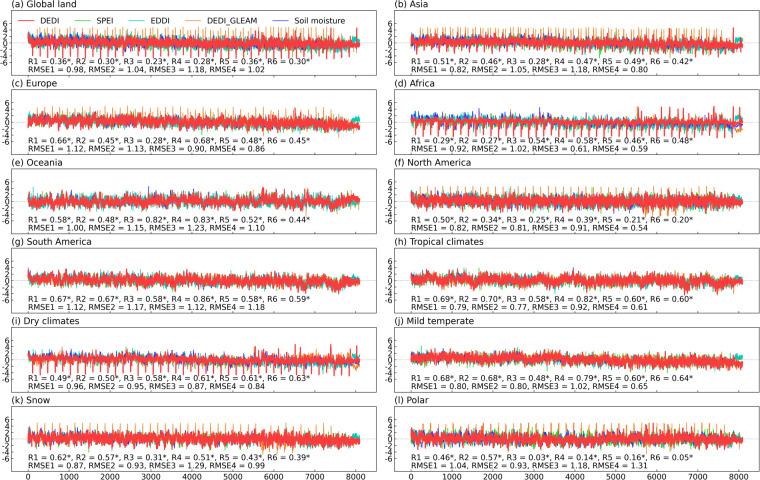
Daily variations of the DEDI with the reference drought indices and soil moisture averaged over (**a**) the global land, (**b**–**g**) six continents, and (**h**–**l**) five major Köppen-Geiger climate zones in the growing season (i.e., March to August) during 1979–2022. The correlation coefficients with asterisks listed at the bottom of each panel indicate significance at a 5% level. R1, R2, and R3 denote the correlation coefficients between DEDI and SPEI, EDDI, and the DEDI_GLEAM, respectively. R4, R5, and R6 denote the correlation coefficients between soil moisture and DEDI, SPEI, and EDDI, respectively. RMSE1, RMSE2, RMSE3, and RMSE4 denote the root mean square errors between DEDI and SPEI, EDDI, the DEDI_GLEAM, and soil moisture, respectively. The correlation coefficients and RMSEs between DEDI and DEDI_GLEAM only refer to their common time period 1980–2022. All series are normalized for visual clarity.

Figure 4 shows the time series of regional averages from global continents and climate zones, which were obtained by estimating the daily average of each year. The DEDI series present similar temporal evolution and general trend to the SPEI and the EDDI series for the past four decades, with significantly high positive correlations (*p* < 0.05) and small root mean square errors. The correlations between the DEDI and the two reference indices largely have coefficients greater than 0.7 on a continental scale, especially with correlations greater than 0.8 over South America, Oceania, and Asia. The DEDI has high correlations with the SPEI and EDDI over the zones with tropical climates (>0.9), dry climates (>0.9), and mild temperate (>0.83), while slightly lower correlations appear over snow (>0.7) and polar (>0.56) zones. The DEDI series agree well with the DEDI_GLEAM at a continental scale, with most correlation coefficients over 0.7. Additionally, the DEDI has significantly high correlations with the DEDI_GLEAM over the zones with tropical climates, dry climates, and mild temperate, while a lower consistency appears over snow and polar zones. Figure 5 further shows the daily changes in each year. The DEDI series have similar daily variations to the reference indices over all regions and present significant positive correlation (*p < *0.05). Nonetheless, more complicated and frequent daily fluctuations tend to degrade the correlation between the DEDI and the reference indices, with correlation coefficients of above 0.5.

In summary, the above validation efforts indicate the high consistency in the temporal evolutions and spatial patterns between the DEDI dataset and the reference drought indices. The DEDI dataset can well capture local and regional dry and wet variations and is able to provide reliable information for drought monitoring and detection.

### Capability of DEDI to capture drying trends

**Fig. 6:**
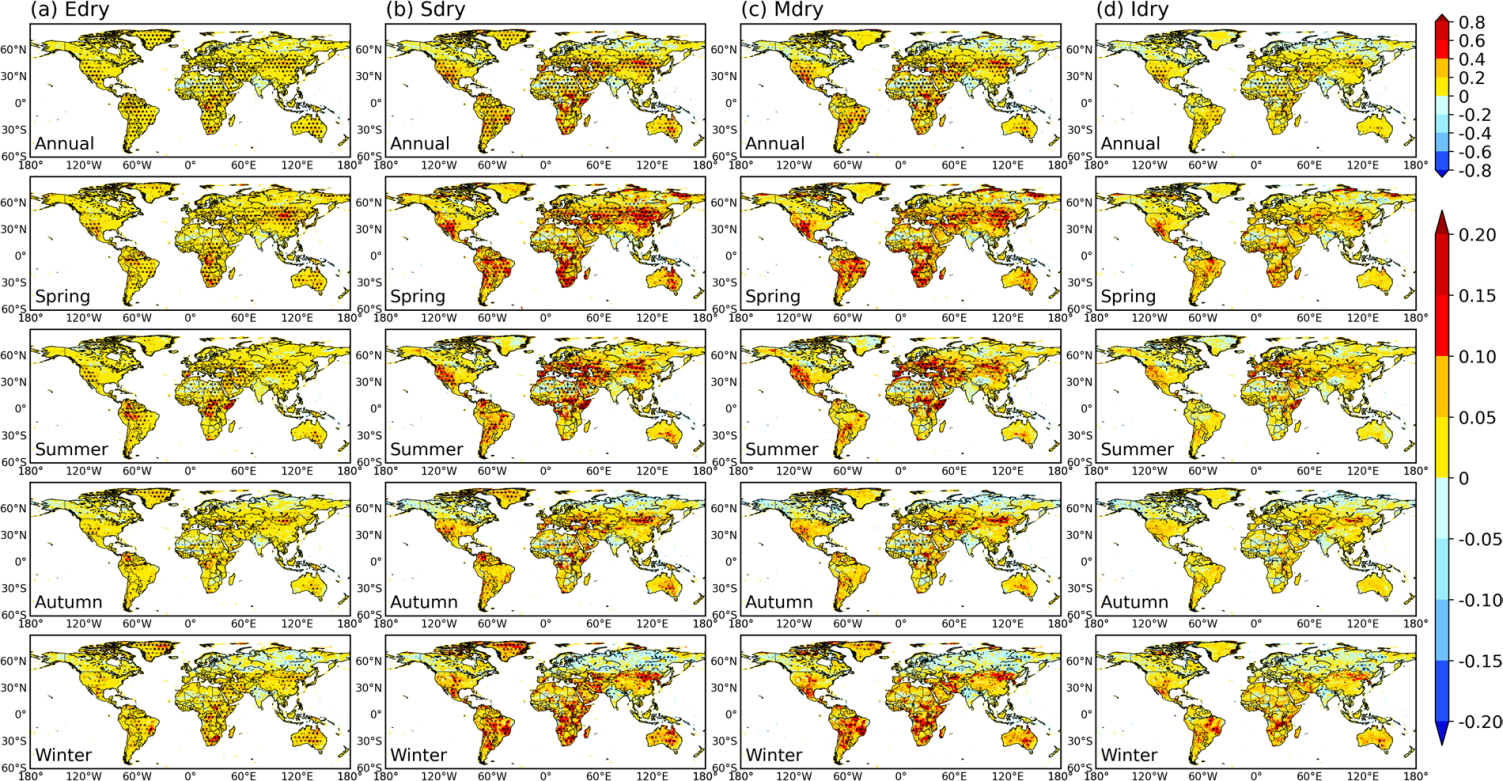
Spatial patterns of trends in the occurrence days of drought with different intensities quantified by the DEDI in different seasons during the period 1979–2021. Edry, Sdry, Mdry, and Idry indicate extreme drought, severe drought, moderate drought, and incipient drought, respectively. The stippled areas indicate the trend being statistically significant at a 5% level. Note that the scale of the first row is different from that of the lower four rows, and the unit of the trend coefficient for all panels is days yr^−1^.

**Fig. 7:**
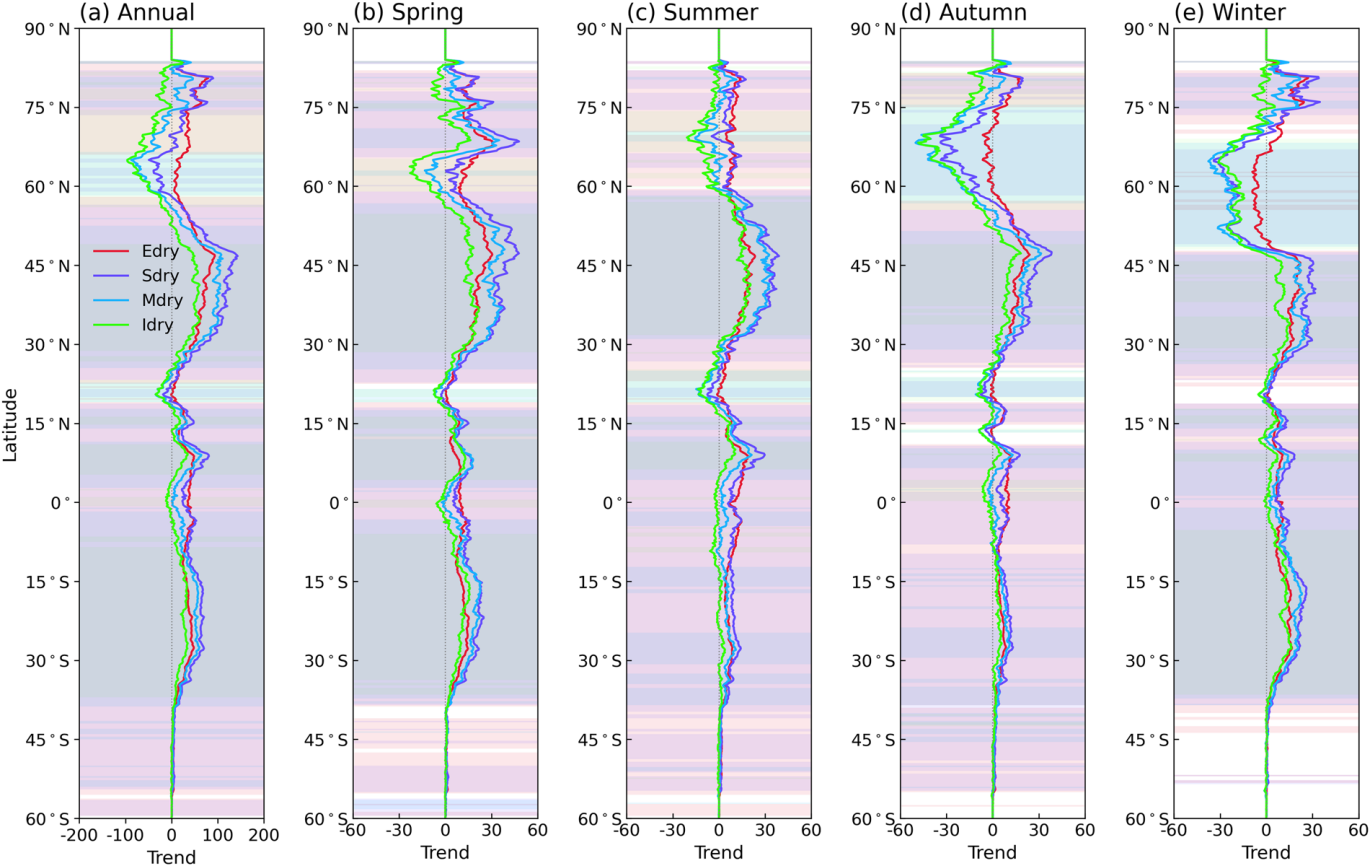
Meridional profiles of trends in the occurrence days of drought with different intensities quantified by the DEDI in different seasons during the period 1979–2021. Edry, Sdry, Mdry, and Idry indicate extreme drought, severe drought, moderate drought, and incipient drought, respectively. The shading denotes the trend being statistically significant at a 5% level. Note that the x-axis scale of (**a**) is different from that of (**b,c**), and the unit of the trend coefficient for all panels is days yr^−1^.

**Fig. 8:**
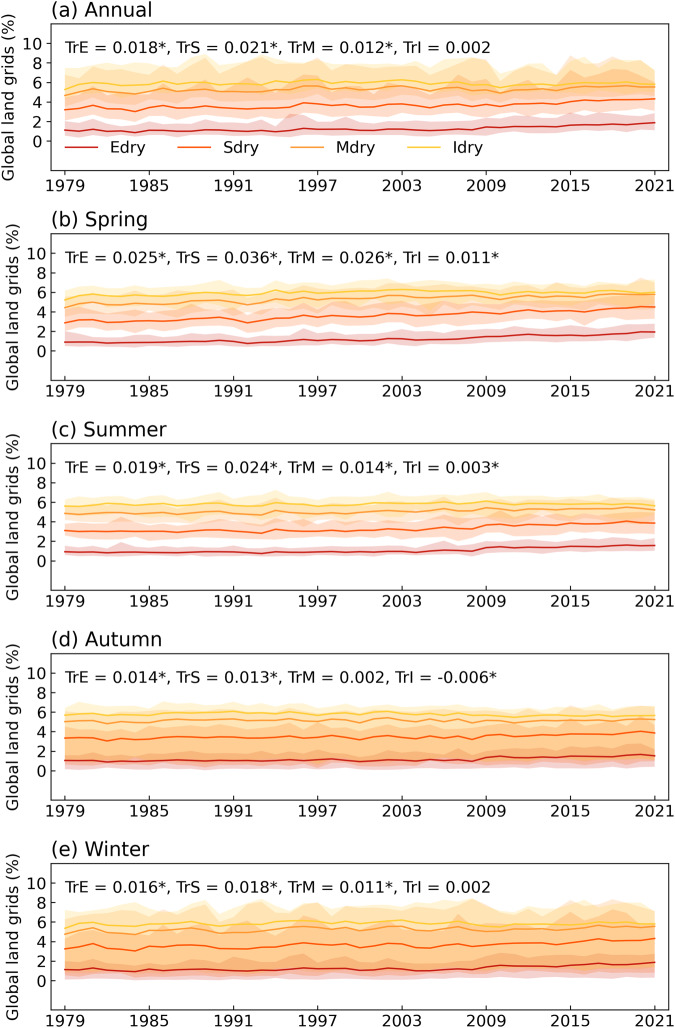
Temporal variations of the proportion of global land grids occurring drought with different intensities quantified by the DEDI in different seasons during the period 1979–2021. Edry, Sdry, Mdry, and Idry indicate extreme drought, severe drought, moderate drought, and incipient drought, respectively. The solid lines show the mean daily proportion for each year, and the shading with lower and upper bounds denotes the minimum and maximum daily proportions. TrE, TrS, TrM, and TrI indicates the trend coefficients of occurring Edry, Sdry, Mdry, and Idry, respectively. The * denotes the trend being statistically significant at a 5% level.

We further assess the DEDI skills in characterizing recent drying trends through the occurrence days of drought with different intensities (i.e., extreme drought, severe drought, moderate drought, and incipient drought). Figure 6 shows the trend of occurring drought categories with different intensities at each pixel over the past 40 years. Almost all drought categories present increasing tendency over most areas of global land, especially for severe and moderate droughts, which have larger positive trend coefficients. Significant increases in drought conditions are mainly pronounced over the regions of central Asia, southwestern North America, southern South America, Africa, southern Europe, and central Australia, where droughts occur with an increase of more than 1 day per year. Among all seasons, almost the entire global land areas in spring and summer have experienced more frequent occurrence of drought in recent decades, while there is a wetting tendency in winter and autumn in most of mid- and high-latitude zones of the northern hemisphere. The meridional profiles (Fig. 7) further clarify these features. The increased trend coefficients appear in all drought categories as proceeding northward from the southern hemisphere, presenting the strongest drying tendency in middle latitudes of the northern hemisphere. At higher northern latitudes, dry and wet conditions exhibit relatively frequent latitude and seasonal fluctuations. More specifically, autumn and winter first show wetting tendency and then drying tendency at latitudes above 70 °N, while spring and summer primarily show drying tendency across the latitudes. In addition, from the time series of the proportion of global land grids experiencing drought with different intensities (Fig. 8), the occurrence of extreme drought tends to have the least days, followed by severe drought, moderate drought, and incipient drought. However, extreme drought, severe drought, and moderate drought have occurred more frequently in recent decades, with significantly increased positive trend coefficients passing a 5% level, especially in spring and summer. Severe drought shows the largest trend coefficients, and extreme drought follows. These results are generally in accordance with previous studies^2–5^.

### Performance of DEDI in characterizing water availability in relation to ecosystems

The evapotranspiration deficit in DEDI considers not only the potential of atmospheric evaporation (PET), but also explicitly accounts for vegetation biotic (transpiration) and abiotic (evaporation) processes. Therefore, DEDI has the potential to serve as a promising tool in detecting ecology- or agriculture-related droughts. Previous studies^21,29,30,56^ using monthly vegetation indices (e.g., leaf area index and normalized difference vegetation index) have already revealed the better performance of the drought indices incorporating AET and PET in detecting the drought impacts on vegetation ecosystem. Here, we assess such ability of the DEDI dataset through comparisons with daily soil moisture and vapor pressure deficit data. Under global warming, soil moisture anomalies largely act as a signal to reflect the impacts of climate change and human activities on terrestrial environments^57,58^ and play an important role in regulating local dry and wet regimes^52,59^. Vapor pressure deficit is a critical variable in determining plant photosynthesis and serves as an important driver of the drought-related vegetation delines^60^. Therefore, soil moisture and vapor pressure deficit are good indications to link vegetation ecosystems and atmospheric dry and wet variations.

**Fig. 9:**
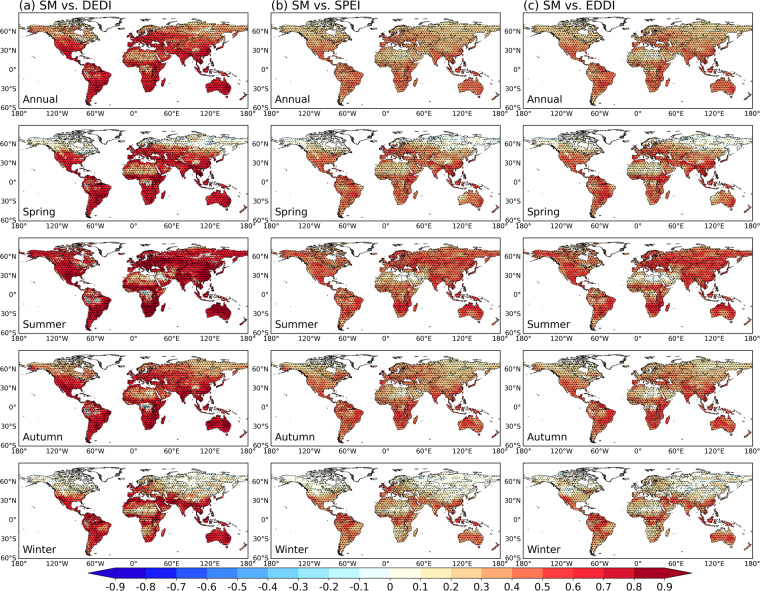
Spatial pattern of correlation coefficients between different daily drought indices (i.e., DEDI in column (**a**), SPEI in column (**b**), and EDDI in column (**c**)) and the soil moisture (abbreviated as SM) in different seasons during the period 1979–2021. The stippled areas indicate the correlation being statistically significant at a 5% level.

**Fig. 10:**
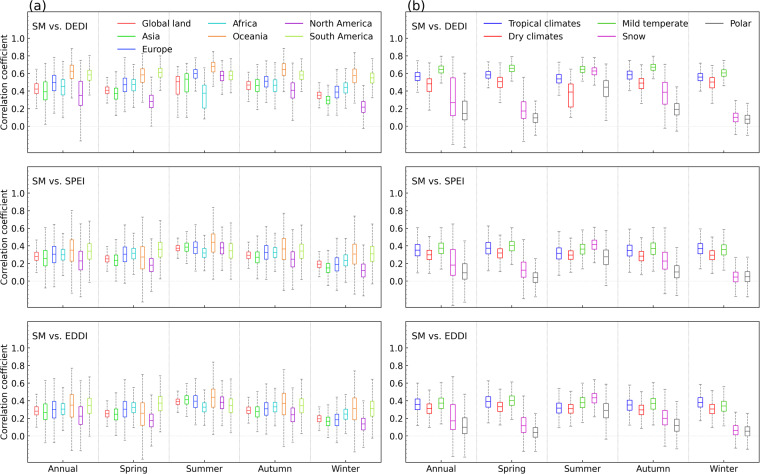
Comparisons of the correlation coefficients between different daily drought indices (i.e., DEDI, SPEI, and EDDI) and the soil moisture (abbreviated as SM) in each grid of the column (**a**) global continental land areas, and the column (**b**) the Köppen-Geiger climate zones in different seasons during the period 1979–2021. The box contains the values from the 25^th^ (bottom) to the 75^th^ (top) percentile, a horizontal line inside the box denotes the median, and the vertical lines denoting whiskers represent the extreme values.

**Fig. 11:**
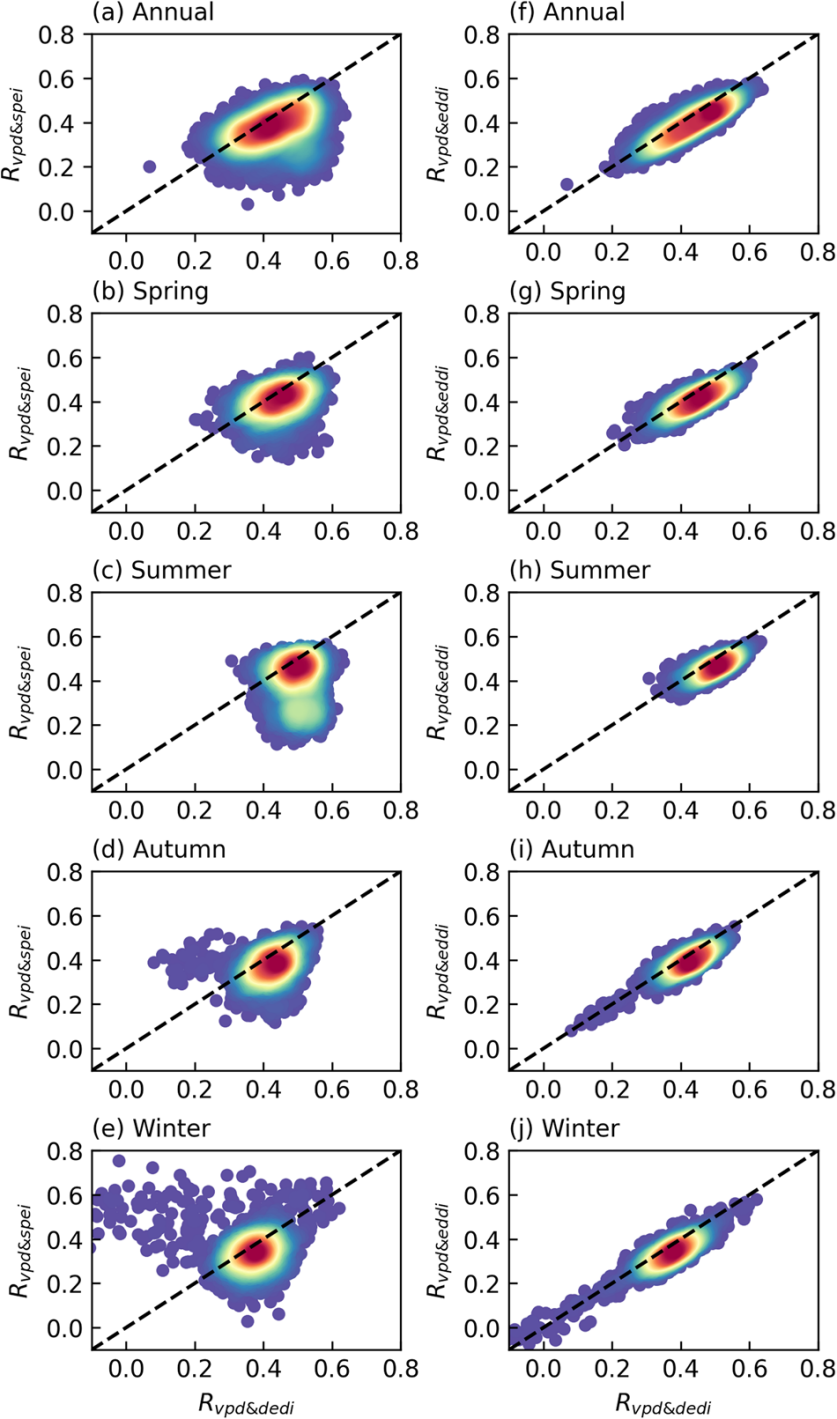
Density scatter comparisons of the correlation coefficients between different daily drought indices (i.e., DEDI, SPEI, and EDDI) and the vapor pressure deficit (abbreviated as VPD) in each grid of the global land in different seasons during the period 1979–2021. *R*_*vpd&dedi*_, *R*_*vpd&spei*_, and *R*_*vpd&eddi*_ indicates the correlations between VPD and DEDI, SPEI, and EDDI, respectively. The colors of the density scatter denote the density of points, i.e., darker red is higher density and darker blue is lower density. The black dashed line is the 1:1 line.

Figures 9, 10 compare the relationship between the DEDI and soil moisture with that between the reference indices and soil moisture, in terms of both spatial and temporal analysis. From the correlation between time series at each pixel of the globe (Fig. 9), all drought indices show overall favourable correspondence with soil moisture. Most areas of global land have statistically significant positive correlation coefficients passing a 5% level, although slightly lower correlation appears in winter. However, the DEDI generally performs the best and has the largest correlation coefficients with soil moisture, compared to the SPEI and the EDDI. Figure 10 shows the series of the daily correlation coefficients of the spatial patterns between the drought indices and soil moisture in global continents and climate zones. The DEDI agrees better with soil moisture, with median correlation greater than 0.4 for almost all continents in different seasons. Furthermore, the DEDI has higher median correlation coefficients greater than 0.5 for all climate zones except snow and polar zones. In comparison, the SPEI and the EDDI have lower correlation with soil moisture. Additionally, from the time series of regional averages in Figs. 4, 5, all drought indices present similar temporal evolutions with soil moisture during the growing season. However, the DEDI has higher correlation with soil moisture almost in all continents and climate zones relative to the SPEI and the EDDI. Figure 11 displays daily cross relationship of the correlations between vapor pressure deficit and drought indices (i.e., DEDI, SPEI, and EDDI). It illustrates that the correlations with vapor pressure deficit are slightly higher for DEDI than for the reference drought indices. All these results demonstrate that the DEDI dataset has potential for use in detection of drought and the impact on ecosystems.

## Usage Notes

The interpretation of the DEDI should be cautious in certain contexts, such as desert areas (e.g., the Sahara Desert) due to excessive evaporation forced by dry air and a lack of observations, forested regions (e.g., the Congo and Amazon basins) due to the ignorance of the signal under vegetation canopy, or frozen conditions (especially at high latitudes) due to the underrepresentation of freeze-thaw parameterization. In addition, the AET used to calculate DEDI is a total evaporation of different components, including bare-soil evaporation, interception loss, and transpiration. Caution should be exercised when the DEDI is validated against soil moisture due to the independency of interception loss from the soil water content, especially during rain events.

## Data Availability

The code used to calculate the DEDI dataset is available via GitHub (https://github.com/XiaZhang1113/Daily-drought-index–DEDI) under the MIT license. The scripts are written with the open-source Python language version 3.8.6 (https://www.python.org/) and the Climate Data Operators (CDO) version 1.9.10 (https://code.mpimet.mpg.de/). Any updates will be published on GitHub.

## References

[1] Hobbins MT (2016). The evaporative demand drought index. Part I: Linking drought evolution to variations in evaporative demand. Journal of Hydrometeorology.

[2] Huang J (2017). Dryland climate change: Recent progress and challenges. Reviews of Geophysics.

[3] Trenberth KE (2014). Global warming and changes in drought. Nature Climate Change.

[4] Ma ZG, Fu CB (2007). Evidences of drying trend in the global during the later half of 20th century and their relationship with large scale climate background. Science China Earth Sciences.

[5] Zhang CC, Yang YT, Yang DW, Wu XC (2021). Multidimensional assessment of global dryland changes under future warming in climate projections. Journal of Hydrology.

[6] Liu WB (2021). Increasing population exposure to global warm-season concurrent dry and hot extremes under different warming levels. Environmental Research Letters.

[7] Hao ZC, AghaKouchak A, Nakhjiri N, Farahmand A (2014). Global integrated drought monitoring and prediction system. Scientific Data.

[8] Bhat GS (2006). The Indian drought of 2002 - a sub-seasonal phenomenon?. Quarterly Journal of the Royal Meteorological Society.

[9] Wong G, Lambert MF, Leonard M, Metcalfe AV (2010). Drought analysis using trivariate copulas conditional on climatic states. Journal of Hydrologic Engineering.

[10] Su BD (2018). Drought losses in China might double between the 1.5 degrees C and 2.0 degrees C warming. Proceedings of the National Academy of Sciences of the United States of America.

[11] Yuan X, Zhang M, Wang LY, Zhou T (2017). Understanding and seasonal forecasting of hydrological drought in the Anthropocene. Hydrology and Earth System Sciences.

[12] Zhang J (2016). Dependence of trends in and sensitivity of drought over China (1961–2013) on potential evaporation model. Geophysical Research Letters.

[13] McKee, T. B., Doesken, N. J. & Kleist, J. *The relationship of drought frequency and duration to time scales in Proceedings of the 8th Conference on Applied Climatology*. 179–183 (American Meteorological Society Boston, MA, 1993).

[14] Wang LY, Yuan X (2018). Two types of flash drought and their connections with seasonal drought. Advances in Atmospheric Sciences.

[15] Brennan, K. E. & Barros, A. P. *The utility of seasonal to interannual climate predictions for water management: A drought forecasting model for the Ohio river basin*. (Helsinki University Technology, 1998).

[16] Vicente-Serrano SM (2012). Performance of drought indices for ecological, agricultural, and hydrological applications. Earth Interactions.

[17] Manzano A (2019). Analysis of the atmospheric circulation pattern effects over SPEI drought index in Spain. Atmospheric Research.

[18] Mishra, A. K. & Singh, V. P. Analysis of drought severity-area-frequency curves using a general circulation model and scenario uncertainty. *Journal of Geophysical Research: Atmospheres* **114**, 10.1029/2008JD010986 (2009).

[19] Richard R. & Heim Jr. A review of twentieth-century drought indices used in the United States. *Bulletin of the American Meteorological Society* **83**, 1149–1165, 10.1175/1520-0477(2002)083<1149:arotdi>2.3.co;2 (2002).

[20] Vicente-Serrano SM, Begueria S, Lopez-Moreno JI (2010). A multiscalar drought index sensitive to global warming: The standardized precipitation evapotranspiration index. Journal of Climate.

[21] Anderson MC (2011). Evaluation of drought indices based on thermal remote sensing of evapotranspiration over the continental United States. Journal of Climate.

[22] Yang, Q., Li, M., Zheng, Z. & Ma, Z. Regional applicability of seven meteorological drought indices in China. *Science China Earth Sciences*, 1–16, 10.1007/s11430-016-5133-5 (2017).

[23] Wang HS (2016). Monitoring winter wheat drought threat in Northern China using multiple climate-based drought indices and soil moisture during 2000-2013. Agricultural and Forest Meteorology.

[24] Mo KC, Lettenmaier DP (2020). Prediction of flash droughts over the United States. Journal of Hydrometeorology.

[25] Meyer SJ, Hubbard KG, Wilhite DA (1993). A crop-specific drought index for corn: I. Model development and validation. Agronomy Journal.

[26] Hunt ED (2014). Monitoring the effects of rapid onset of drought on non-irrigated maize with agronomic data and climate-based drought indices. Agricultural and Forest Meteorology.

[27] Zhang X, Duan YW, Duan JP, Jian DN, Ma ZG (2022). A daily drought index based on evapotranspiration and its application in regional drought analyses. Science China-Earth Sciences.

[28] Zhang, X. *et al*. A daily drought index-based regional drought forecasting using the Global Forecast System model outputs over China. *Atmospheric Research*, 106166, 10.1016/j.atmosres.2022.106166 (2022).

[29] Vicente-Serrano SM (2018). Global assessment of the standardized evapotranspiration deficit index (SEDI) for drought analysis and monitoring. Journal of Climate.

[30] Zhang X (2019). Assessment of an evapotranspiration deficit drought index in relation to impacts on ecosystems. Advances in Atmospheric Sciences.

[31] Hersbach, H. *et al*. ERA5 hourly data on single levels from 1940 to present. *Copernicus Climate Change Service (C3S) Climate Data Store (CDS)*, 10.24381/cds.adbb2d47 (2023).

[32] Hersbach H (2020). The ERA5 global reanalysis. Quarterly Journal of the Royal Meteorological Society.

[33] Martens B (2020). Evaluating the land-surface energy partitioning in ERA5. Geoscientific Model Development.

[34] Sun GH (2020). Analysis of local land-atmosphere coupling in rainy season over a typical underlying surface in Tibetan Plateau based on field measurements and ERA5. Atmospheric Research.

[35] Li M, Wu P, Sexton DMH, Ma Z (2021). Climate Dynamics.

[36] Eltahir EAB, Yeh PJ-F (1999). On the asymmetric response of aquifer water level to floods and droughts in Illinois. Water Resources Research.

[37] Shellito PJ (2016). SMAP soil moisture drying more rapid than observed *in situ* following rainfall events. Geophysical Research Letters.

[38] Muñoz Sabater J (2019). Copernicus Climate Change Service (C3S) Climate Data Store (CDS).

[39] Munoz-Sabater J (2021). ERA5-Land: a state-of-the-art global reanalysis dataset for land applications. Earth System Science Data.

[40] Martens B (2017). GLEAM v3: Satellite-based land evaporation and root-zone soil moisture. Geoscientific Model Development.

[41] Liu WB (2016). A worldwide evaluation of basin-scale evapotranspiration estimates against the water balance method. Journal of Hydrology.

[42] Dembele M (2020). Potential of satellite and reanalysis evaporation datasets for hydrological modelling under various model calibration strategies. Advances in Water Resources.

[43] Miralles DG, van den Berg MJ, Teuling AJ, de Jeu RAM (2012). Soil moisture-temperature coupling: A multiscale observational analysis. Geophysical Research Letters.

[44] Holwerda F, Bruijnzeel LA, Scatena FN, Vugts HF, Meesters A (2012). Wet canopy evaporation from a Puerto Rican lower montane rain forest: The importance of realistically estimated aerodynamic conductance. Journal of Hydrology.

[45] Chen D, Chen HW (2013). Using the Köppen classification to quantify climate variation and change: An example for 1901–2010. Environmental Development.

[46] WMO. WMO guidelines on the calculation of climate normals. *WMO Technical Report* (2017).

[47] Svoboda, M. *et al*. The drought monitor. *Bulletin of the American Meteorological Society* **83**, 1181–1190, 10.1175/1520-0477(2002)083<1181:tdm>2.3.co;2 (2002).

[48] Chen HL (2022). Uncertainties in partitioning evapotranspiration by two remote sensing-based models. Journal of Hydrology.

[49] Begueria S, Vicente-Serrano SM, Reig F, Latorre B (2014). Standardized precipitation evapotranspiration index (SPEI) revisited: parameter fitting, evapotranspiration models, tools, datasets and drought monitoring. International Journal of Climatology.

[50] Ma B, Zhang B, Jia LG, Huang H (2020). Conditional distribution selection for SPEI-daily and its revealed meteorological drought characteristics in China from 1961 to 2017. Atmospheric Research.

[51] Wang Q (2021). A multi-scale daily SPEI dataset for drought characterization at observation stations over mainland China from 1961 to 2018. Earth System Science Data.

[52] Li MX, Wu PL, Ma ZG (2020). A comprehensive evaluation of soil moisture and soil temperature from third-generation atmospheric and land reanalysis data sets. International Journal of Climatology.

[53] Zhang X, Duan J, Cherubini F, Ma Z (2023). Zenodo.

[54] Qin Y (2017). Assessment of reanalysis soil moisture products in the permafrost regions of the central of the Qinghai–Tibet Plateau. Hydrological Processes.

[55] Wang C, Yang K (2018). A new scheme for considering soil water-heat transport coupling based on Community Land Model: Model description and preliminary validation. Journal of Advances in Modeling Earth Systems.

[56] Vicente-Serrano SM (2013). Response of vegetation to drought time-scales across global land biomes. Proceedings of the National Academy of Sciences.

[57] D’Odorico P, Porporato A (2004). Preferential states in soil moisture and climate dynamics. Proceedings of the National Academy of Sciences of the United States of America.

[58] Seneviratne SI (2010). Investigating soil moisture-climate interactions in a changing climate: A review. Earth-Science Reviews.

[59] Koster RD (2004). Regions of strong coupling between soil moisture and precipitation. Science.

[60] Yuan W (2019). Increased atmospheric vapor pressure deficit reduces global vegetation growth. Science Advances.

